# The Mycobacterial DNA Methyltransferase HsdM Decreases Intrinsic Isoniazid Susceptibility

**DOI:** 10.3390/antibiotics10111323

**Published:** 2021-10-29

**Authors:** Xinling Hu, Xintong Zhou, Tong Yin, Keyu Chen, Yongfei Hu, Baoli Zhu, Kaixia Mi

**Affiliations:** 1 CAS Key Laboratory of Pathogenic Microbiology and Immunology, Institute of Microbiology, Chinese Academy of Sciences, Beijing 100101, China; huxl@im.ac.cn (X.H.); zhouxt@im.ac.cn (X.Z.); yintong0227@126.com (T.Y.); tiberius123@163.com (K.C.); zhubaoli@im.ac.cn (B.Z.); 2 Savaid Medical School, University of Chinese Academy of Sciences, Beijing 101408, China; 3 State Key Laboratory of Animal Nutrition, College of Animal Science and Technology, China Agricultural University, Beijing 100193, China; huyongfei@cau.edu.cn

**Keywords:** DNA methyltransferase, HsdM, isoniazid, *Mycobacterium bovis* BCG, drug susceptibility

## Abstract

Tuberculosis, caused by the pathogen *Mycobacterium tuberculosis*, is a serious infectious disease worldwide. Multidrug-resistant TB (MDR-TB) remains a global problem, and the understanding of this resistance is incomplete. Studies suggested that DNA methylation promotes bacterial adaptability to antibiotic treatment, but the role of mycobacterial HsdM in drug susceptibility has not been explored. Here, we constructed an inactivated *Mycobacterium bovis* (BCG) strain, Δ*hsdM*. Δ*hsdM* shows growth advantages over wild-type BCG under isoniazid treatment and hypoxia-induced stress. Using high-precision PacBio single-molecule real-time sequencing to compare the Δ*hsdM* and BCG methylomes, we identified 219 methylated HsdM substrates. Bioinformatics analysis showed that most HsdM-modified genes were enriched in respiration- and energy-related pathways. qPCR showed that HsdM-modified genes directly affected their own transcription, indicating an altered redox regulation. The use of the latent Wayne model revealed that Δ*hsdM* had growth advantages over wild-type BCG and that HsdM regulated *trcR* mRNA levels, which may be crucial in regulating transition from latency to reactivation. We found that HsdM regulated corresponding transcription levels via gene methylation; thus, altering the mycobacterial redox status and decreasing the bacterial susceptibility to isoniazid, which is closely correlated with the redox status. Our results provide valuable insight into DNA methylation on drug susceptibility.

## 1. Introduction

Tuberculosis (TB) is a chronic wasting infectious disease that causes infections of various tissues and organs after *Mycobacterium tuberculosis* (Mtb) is inhaled, and then spreads through the respiratory tract. According to the Global Tuberculosis Report issued by the World Health Organization in 2019 [1], the number of TB-infected patients has been relatively stable in recent years, with approximately 10 million new cases annually worldwide. In 2018, TB caused approximately 1.5 million deaths and is among the top ten causes of human deaths. TB causes most infection-related deaths primarily because of antimicrobial resistance. Understanding how Mtb mutates and evolves in the host to produce drug resistance will provide theoretical guidance for improving TB treatment and control strategies. Mtb is genetically static, with a low mutation rate, and evolves via single nucleotide polymorphisms [2,3]. However, under continued chemotherapy, a fraction of Mtb eventually develops complete antibiotic resistance [4]. It remains unclear how this genetically stable organism adapts so quickly to antibiotic treatments and infected host’s immune pressures such as reactive oxygen species, which are products of the host’s innate immune responses induced by infected Mtb [5].

Recent studies have shown that bacterial epigenomics is an important and newly rising field for genetic and phenotypic analysis of microbial diversity, gene regulation and evolution. Modifying DNA to regulate gene expression facilitates physiological adaptation to new environments without needing to drastically alter the genome. Previous studies of bacterial methylation have shown that DNA methylation promotes bacterial adaptability in infected hosts [6,7] and to antibiotic treatments [8]. These studies suggested that environmental changes (such as pathogens infecting the host) lead to selection of a well-adjusted subgroup. That is, this phenotypic plasticity helps bacteria quickly adapt to changing environmental pressures. Studies have shown that the different evolutionary lineages of Mtb come from different geographical regions [9], and genetic variations in these TB subgroups affect the evolution of drug resistance [10].

We speculate that DNA methylation is a feasible but poorly understood mechanism of Mtb phenotypic variation. Mtb has three DNA methyltransferases (MTases), MamA, MamB and HsdM, which target different DNA sequence motifs of N6-adenine methylation [11,12,13]. The bacterial methylome has been linked to antibiotic stress survival [8], indicating that DNA methylation may play important roles in antibiotic susceptibility. Most methylation studies on Mtb have been conducted on strains collected from clinical samples, and the MTases in these strains have complex backgrounds with varying numbers of MTases and different degrees of mutated sites; thus, conclusions from these studies are conflicting [13,14].

Here, we characterize the biological function of an orphan MTase, HsdM, in *Mycobacterium bovis* BCG Pasteur (BCG). Using specialized transduction, we constructed Δ*hsdM* and showed that Δ*hsdM* decreased the bacterial susceptibility to isoniazid (INH) compared with that of its parental strain, BCG. We used high-precision PacBio single-molecule real-time (SMRT) sequencing technology to compare the Δ*hsdM* and BCG methylomes. We identified 219 methylated HsdM substrates, of which, 192 were located in open reading frames (ORFs), and 28 were located upstream of ORFs. Bioinformatics analysis showed that most HsdM-modified genes were enriched in respiration- and energy-related pathways. qPCR showed that HsdM modification regulated the transcription level of its modified DNA, suggesting that HsdM-mediated changes in mRNA levels may be related to redox regulation. Using the latent Wayne model, we show that Δ*hsdM* had a growth advantage over wild-type BCG under hypoxic conditions and that HsdM regulated *trcR* mRNA levels, which was predicted to be a key regulator of transition from latency to reactivation. Our results showed that HsdM regulated the corresponding transcription levels via gene methylation, leading to changes in the mycobacterial redox status, thereby affecting mycobacterial susceptibility to INH, which was closely correlated with the mycobacterial redox status.

## 2. Results

### 2.1. DNA Methyltransferase HsdM Decreased INH Susceptibility in Mycobacteria

Previous studies have shown that MTases mediate various functions, including antibiotic stress responses [8,15,16,17]. Mycobacterial DNA methylation is complex. Mtb has three known MTases, MamA (Rv3263), HsdM (Rv2756c) and MamB (Rv2024c), which modify distinct DNA sequence motifs [12,13]. Mycobacteria from different lineages contain different numbers of DNA MTases from different sources with different mutation sites [11,12,13]. These complexities make it difficult to identify the functions of these MTases. The clinical isolates containing different numbers of MTases and different degrees of mutated sites [13,14] are difficult to explore the biological functions of HsdM. Here, we used *M. bovis* BCG Pasteur (BCG) to assess how HsdM functioned in antibiotic susceptibility. HsdM in BCG is similar to HsdM in Mtb H37Rv. BCG_2772c from BCG is equivalent to Rv2756c from *M. tuberculosis* strain H37Rv with 99.8% identity (Mycobrowser website https://mycobrowser.epfl.ch/genes (accessed on 12 August 2021) and KEGG website https://www.kegg.jp (accessed on 12 August 2021)). Additionally, BCG contains three intact DNA MTases. We examined whether HsdM affected DNA methylation via MamA and MamB.

**Figure 1:**
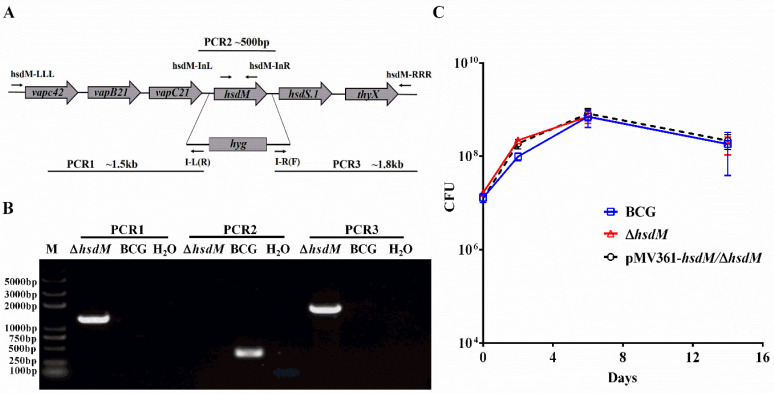
HsdM did not impact mycobacterial growth in vitro. (**A**) Genomic organization of the *hsdM* gene locus. Large arrows represent coding genes in their orientation. Small arrows represent the primer pair used for corresponding PCR. (**B**) PCR to confirm the knockout *hsdM* (Δ*hsdM*) strains. The replacement gene, hygromycin-coding gene (*hyg*), is also shown. The PCR products of the upstream (PCR1) and downstream (PCR3) regions of *hsdM* using the primers pairs LLL/I-L(R) and I-R(F)/RRR, respectively. The PCR product of the gene fragment in *hsdM* using primers hsdM-InL and hsdM-InR (PCR2). (**C**) Growth curves of BCG, Δ*hsdM* and pMV361-*hsdM*/Δ*hsdM* strains in 7H9 medium. Data presented the means ± standard deviations (SD) from three independent experiments.

First, we constructed an *hsdM* knockout in BCG using specialized transduction [18]. We cloned approximately 1 kb fragments both upstream and downstream of *hsdM* via PCR and constructed an allelic exchange vector on phAE159 [18,19]. Colonies carrying *hyg* to replace *hsdM* were confirmed via PCR (Figure 1A,B). The corresponding complementary strain pMV361-*hsdM*/Δ*hsdM* was constructed. The in vitro growth kinetics of Δ*hsdM* in Middlebrook 7H9 supplemented with albumin–dextrose–saline (ADS) were comparable to those of wild-type BCG and the complementary strain for up to 14 days of incubation (Figure 1C). No differences were observed throughout the entire growth process, indicating that HsdM did not affect mycobacterial growth in vitro in ADS-supplemented Middlebrook 7H9.

**Figure 2:**
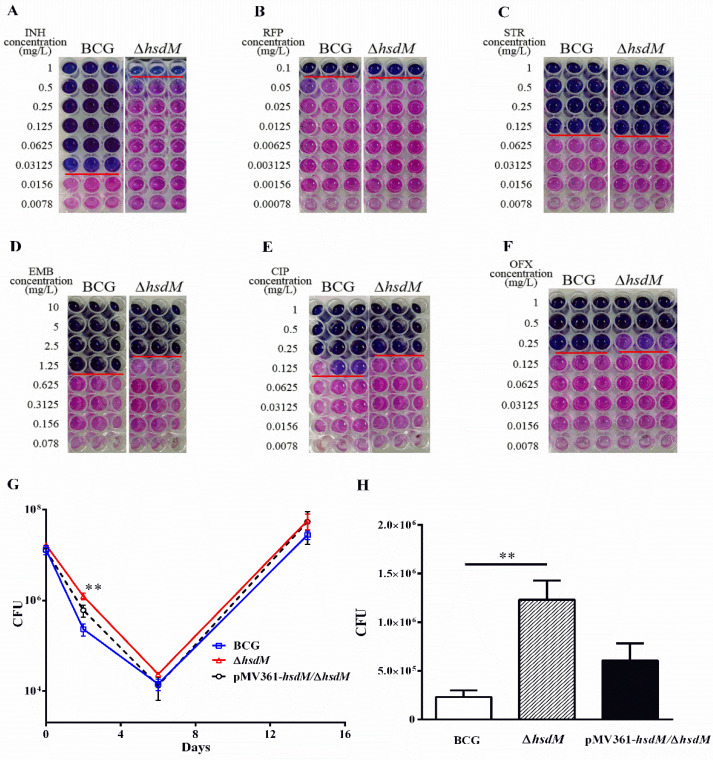
HsdM affected drug susceptibility in BCG. (**A**–**F**) MICs of INH, RFP, EMB, STR, OFX and CIP on microplates in BCG and Δ*hsdM*. Blue wells indicate no growth owing to drug inhibition; pink wells indicate growth. (**G**) Growth curves of BCG, Δ*hsdM* and pMV361-*hsdM*/Δ*hsdM* strains treated with 0.25 mg/L INH. (**H**) The number of surviving cells was examined by monitoring the colony-forming units (CFUs) for 2 days. Data are presented as the mean ± standard deviation of three independent replicates. The figure presents the results of three biological replicates. ** *p* < 0.01.

Next, we selected antibiotics used for the clinical treatment of TB, including isoniazid (INH), rifampicin (RFP), streptomycin (STR), ethambutol (EMB), ciprofloxacin (CIP) and ofloxacin (OFX), compared the susceptibility of Δ*hsdM* and wild-type BCG (Figure 2). Strikingly, Δ*hsdM* showed a marked resistance to INH compared with that of BCG (Figure 2A). The minimum inhibitory concentration (MIC) of Δ*hsdM* to INH was ~32-fold higher than that of the wild-type BCG exposed to INH. No differences in antibiotic susceptibility between Δ*hsdM* and wild-type BCG were detected in any other antibiotics (Figure 2B–F). We, at first, performed antibiotic susceptibility testing of three mycobacterial strains (BCG, Δ*hsdM* and pMV361-*hsdM/*Δ*hsdM*) treated with INH at the indicated concentrations and showed the growth advantage of Δ*hsdM* was partly reduced by complementation with an integrated copy of *hsdM*, pMV361-*hsdM/*Δ*hsdM* (Supplementary Material Figure S1). To further confirm the effect of HsdM on INH susceptibility, we performed the drug exposure experiments in the presence of 0.25 mg/L INH (8 × MIC of INH) to compare the growth rates among strains BCG, Δ*hsdM* and pMV361-*hsdM*/Δ*hsdM.* Consistent to previous studies [20,21,22], we observed the typical biphasic killing curve of these three mycobacterial strains (Figure 2G). All three of these strains showed typical INH-killing growth curves after INH treatment; that is, the INH treatment arrested the mycobacterial growth for up to 6 days. After reaching the lowest point, the mycobacterial tolerance to INH began to increase (Figure 2G). On days 2–3 after INH treatment, Δ*hsdM* exhibited a significant growth advantage (Figure 2H), which was partly reduced by complementation with an integrated copy of *hsdM* constitutively expressed and driven by the mycobacterial promoter, pMV361-*hsdM*/Δ*hsdM*. Thus, HsdM affected the mycobacterial susceptibility to INH likely because HsdM methylation-modified DNA affected transcription, which in turn affected INH susceptibility.

### 2.2. Bioinformatic Analysis of the HsdM Substrate via Whole-Genome Sequencing

To verify our hypothesis that HsdM affected susceptibility to INH via its methylated DNA, the three mycobacterial strains BCG, Δ*hsdM* and pMV361-*hsdM/*Δ*hsdM* were sequenced via PacBio SMRT sequencing [23], which enables directly detecting methylated DNA at the genomic level. A previous study showed that the GATC methylome was stable, and drug treatment did not affect the adenine methylome [8]; thus, we did not examine the mycobacterial methylome after drug treatment. Three strains were cultured on 7H10 plates supplemented with ADS for 10 days, and all colonies growing on the plates were collected. Genomic DNA was extracted and sequenced using PacBio SMRT sequencing. The average sequencing coverages of BCG, Δ*hsdM* and pMV361-*hsdM/*Δ*hsdM* were approximately 133.79×, 156.51× and 219.69×, respectively. Similar to a previous study [12], the sequencing results provided general bioinformation, including the respective genomic sizes (~4.345–4.352 Mb), the number of ORFs (~4069–4102) and the GC% (65.6%). Adenine MTases have been shown to target distinctive DNA sequence motifs, of which, MamA, MamB and HsdM target the corresponding motifs CTCCAG, CACGCAG and GATN_4_RTAC, respectively [11,12]. The percentage of GATN_4_RTAC sites methylated by HsdM in BCG was 72.5%; no methylated GATN_4_RTAC sites were detected in Δ*hsdM* (Table 1). The percentage of the methylated GATN_4_RTAC sites was 58.7% in the complementary pMV361-*hsdM/*Δ*hsdM* strain, indicating that a single integrated copy of *hsdM* expressed from a mycobacterial promoter could at least partially complement the methylation function of HsdM. Most of the CTCCAG (99.3%) and CACGCAG (99.8%) were methylated by the corresponding MamA and MamB in BCG. Additionally, the motifs of both MamA and MamB were almost fully methylated (99.1% and 100%, respectively) in Δ*hsdM.* Similar to the methylation profiles modified by MamA and MamB in BCG and Δ*hsdM*, approximately 99–100% of the methylated motifs were modified in pMV361-*hsdM/*Δ*hsdM.* Hence, HsdM did not affect DNA methylation via MamA and MamB.

The SMRT sequencing results for BCG, Δ*hsdM* and pMV361-*hsdM/*Δ*hsdM* revealed 489 methylated sites, of which 430 were located in the 192 ORFs, and 59 were located in noncoding gene regions, including 28 downstream genes (Supplementary Material Tables S1 and S2). One substrate, Rv1522c, had modification sites both in and upstream of the gene.

**Figure 3:**
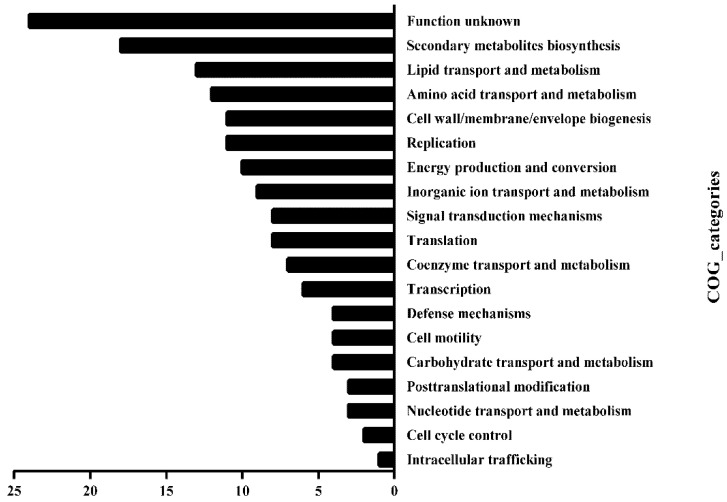
Functional classification of HsdM substrates in BCG. Substrate gene functions were classified by comparing them in the COG database.

We, then, investigated the Clusters of Orthologous Groups (COG) functional category of those substrates. The methylated substrates of HsdM included 219 genes (Tables S1 and S2), of which, 158 were successfully annotated by COG and fell into 19 classification categories, including replication, recombination and repair, transcription, translation, ribosomal structure and biogenesis, lipid transport and metabolism and carbohydrate transport metabolism (Figure 3). Manually searching the biofunctions of these substrates revealed that 56 methylated substrates of HsdM (~25%) were involved in the respiration pathway (Table S3), and the bioinformatic analysis implied that HsdM-mediated modification was linked to cellular redox regulation or energy metabolism. Previous studies have shown that bacterial antibiotic susceptibility is associated with the redox status [24,25]. Therefore, after combining the experimental results and bioinformatics analysis of the HsdM substrates, we speculated that methylation by HsdM affects the transcription level of the corresponding substrates; thus, affecting the redox state of the cell and affecting its susceptibility to INH, a prodrug with redox-mediated activation [26,27].

### 2.3. HsdM Regulated Gene Expression of Its Substrates

**Figure 4:**
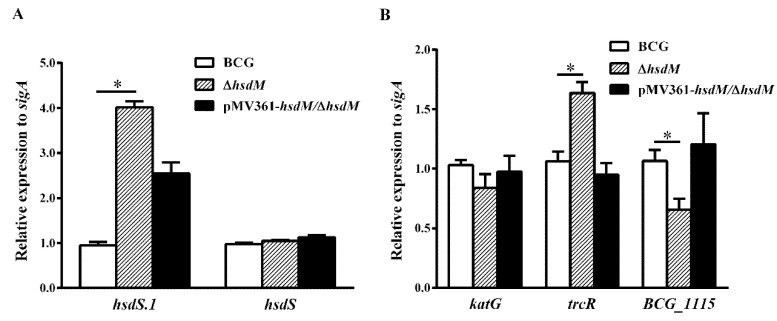
Genes differentially expressed in ∆*hsdM*. Expression of each gene was determined by qPCR in the ∆*hsdM* strain and its parental strain. (**A**) Relative expressions of *hsdS* and *hsdS.1.* * *p* < 0.05. (**B**) qPCR analysis of the mRNA expression of *katG, trcR* and *BCG_1115*.

To verify whether HsdM methylation affected the transcriptional level of its substrates, resulting in different mycobacterial redox statuses and affecting the susceptibility to INH, we designed qPCR primers to compare the mRNA levels of all identified HsdM substrates between BCG and ∆*hsdM.* Under the detection conditions of all HsdM substrates, 17 ORFs were significantly downregulated, and 41 were significantly upregulated in ∆*hsdM* compared with those in BCG (Table S1). The qPCR examination of the cis-regulation of DNA methylation revealed that nine methylation sites upstream caused statistically different BCG and ∆*hsdM* expressions in the corresponding genes (Table S2). Consistent with a previous study [14], *hsdS.1* (equivalent to *rv2755c* in Mtb) expression was significantly upregulated in the knockout strain (Figure 4A), suggesting that its regulation was associated with *hsdM* (equivalent to *rv2756c* in Mtb). Conversely, *hsdS* (equivalent to *rv2761c* in Mtb) was unchanged (Figure 4A). Additionally, the two-component system response regulator, TrcR (equivalent to Rv1033c in Mtb), which is thought to help bacteria adapt to the infection environment in the host and switch from latency to reactivation [28], was upregulated in ∆*hsdM* compared with that in the wild-type. This switch process is closely linked to redox regulation [25]. Moreover, a study showed that TrcR represses BCG_1115 (equivalent to Rv1057 in Mtb), a putative surface antigen [29]. The qPCR analysis showed that *BCG_1115* expression levels were downregulated in ∆*hsdM* compared with those in the wild-type, which was consistent with the previously reported negative regulation of *BCG_1115* (equivalent to *rv1057* in Mtb*)* by TrcR (Figure 4B).

To rule out that the reduced susceptibility of ∆*hsdM* to INH compared with that of BCG was due to differential *katG* expression, we examined the expression levels of *katG* in ∆*hsdM* and BCG via qPCR. The qPCR analysis showed that *katG* mRNA levels in ∆*hsdM* were not significantly reduced (~0.84 ± 0.08-fold) compared with those of the parental strain, BCG (Figure 4B). These results suggested that HsdM methylation affected gene expression in the reduced INH susceptibility of ∆*hsdM* compared with that of BCG.

### 2.4. HsdM Deletion Increases Survival of BCG during Hypoxia

**Figure 5:**
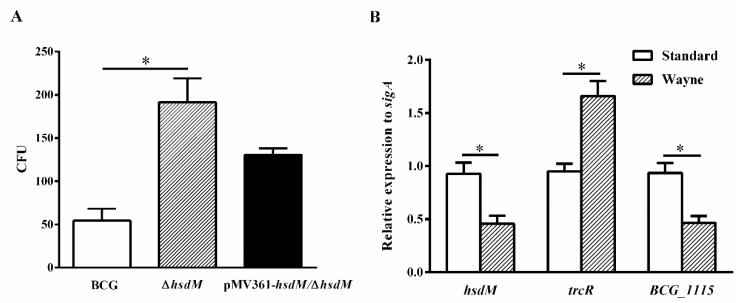
*HsdM* deletion increased survival of BCG under hypoxia. (**A**) CFUs of the ∆*hsdM*, wild-type BCG and complementary strain pMV361-*hsdM*/∆*hsdM* were detected under the latent Wayne model. (**B**) qPCR analysis of the mRNA expressions of *hsdM*, *trcR* and *BCG_1115* in BCG between the latent Wayne model and standard culture. * *p* < 0.05.

To further confirm the role of HsdM in oxidative stress responses, we constructed a latent Wayne model [30] and examined the growth kinetics and expression levels of related genes of BCG, ∆*hsdM* and pMV361-*hsdM/*∆*hsdM.* At 10 days postinoculation, all strains were collected and plated on 7H10 media supplemented with ADS. When the methylene blue, an oxygen concentration indicator, became colorless, it indicated that the O_2_ concentration dropped to 0. ∆*hsdM* exhibited a significant growth advantage over BCG (Figure 5A). Growth kinetics of the complemented strains pMV361-*hsdM/*∆*hsdM* and BCG did not differ, indicating that ∆*hsdM* had a clear growth advantage relative to that of wild-type BCG under hypoxic conditions. Under these conditions, the relative *hsdM* mRNA levels decreased by 0.49 ± 0.06-fold, and compared with those under standard culture conditions in BCG, the relative *trcR* mRNA levels increased by 1.75 ± 0.11-fold. Additionally, *BCG_1115* was decreased by 0.50 ± 0.05-fold (Figure 5B). Hence, HsdM-mediated modification was linked to redox regulation.

## 3. Discussion

Here, we showed that mycobacterial HsdM, an adenine methyltransferase, altered the transcription levels of the corresponding substrates by methylating its own substrates, thereby reducing the mycobacterial susceptibility to INH. Furthermore, HsdM deletion increased mycobacterial survival under hypoxia. Our results indicated that HsdM-mediated DNA methylation alters mycobacterial sensitivity to INH.

To explore the biological role of HsdM, we took advantage of antibiotics as chemical probes to screen the difference between the wild-type strain BCG and the mutant strain ∆*hsdM* (Figure 2A–F). Both previous studies and our studies linked isoniazid action with redox homeostatic [21,31,32]. We observed that the *hsdM* knockout in BCG showed that ∆*hsdM* had remarkable resistance to INH compared with that of BCG (Figure 2A), and complementary HsdM partially abrogated INH resistance in ∆*hsdM* (Figure 2G,H). We hypothesized that HsdM affects the transcriptional levels of its substrates, which in turn affects the oxidation reduction in the bacteria. To prove our hypothesis, we compared the methylomes of those three strains (BCG, Δ*hsdM* and pMV361-*hsdM/*Δ*hsdM)* using high-precision PacBio single-molecule real-time (SMRT) sequencing technology.

SMRT technology has been used to identify methylated DNA sequences in bacteria, including *Mycobacterium* [8,12,13,14,33]. In mycobacteria, HsdM is predicted to be an orphan that lacks cognate restriction enzymes [34]. This means that unmethylated HsdM-modified DNA sequences cannot be degraded; that is, the HsdM-modified motifs are stable regardless of whether they are methylated or unmethylated. Consistent with a previous study [12,13,14], the percentage/ratio of methylated modification by HsdM was lower (67–69%), indicating that methylation by HsdM is limited to specific biological functions. As predicted, HsdM substrates were enriched in the respiration pathway (Table S3), whereas the MamA and MamB substrates were not enriched in specific pathways. The bioinformatic analysis of 25% of HsdM substrates presenting in the respiration pathway supported our hypothesis that biological function of HsdM links to redox homeostatic regulation.

Recent studies on the bacterial GATC methylome suggested that MTases regulate transcription [12,13,14,35,36]. In Mtb, more HsdM motifs than MamA and SigA motifs are located in ORFs [14], suggesting that the biological functions of HsdM modification differ from those of MamA and SigA. Here, we showed that HsdM methylated 88% of sites in ORFs. Further assays showed that the expression levels of most motifs tended to be upregulated in ∆*hsdM* (Table S1). Thus, HsdM-methylated encoding genes could directly affect their own transcription.

Through a comparison of different methylomes in BCG and ∆*hsdM,* we identified 219 HsdM substrates and confirmed via qPCR (Table S1). In particular, *trcR*, a two-component system member, was identified as a HsdM substrate (Table S1). The *trcR* mRNA levels were upregulated in ∆*hsdM* compared with those in BCG (Figure 4). Because TrcR is important in reactivation from latency [28], a process being linked to the redox status change. Using a Wayne model of hypoxia, we also showed increased *trcR* mRNA levels under hypoxic conditions, compared with standard growth (growth in 7H9 medium) (Figure 5B). Additionally, ∆*hsdM* exhibited a significant growth advantage over BCG (Figure 5A). Thus, HsdM is important for fitness during hypoxia. Similar to a previous study [31], we found a relationship between the latent gene, *trcR*, and drug resistance, which, thus, requires further study. MamA has also been associated with hypoxia [11]. Further studies are needed to determine how the synergistic effect of MamA and HsdM on genome methylation affects mycobacterial antibiotic susceptibility.

## 4. Materials and Methods

### 4.1. Bacterial Strains and Culture Conditions

In this study, *Mycobacterium bovis* BCG Pasteur (BCG) was used to assess HsdM functions in antibiotic susceptibility. Mycobacterial strains were grown in 7H9 medium comprising Middlebrook 7H9 medium (Becton Dickinson, Sparks, MD, USA) supplemented with 10% ADS (5% *w/v* bovine serum albumin fraction V, 2% *w/v* D-dextrose, 8.1% *w/v* NaCl), 0.5% *v/v* glycerol and 0.05% *v/v* TWEEN 80. The ∆*hsdM* mutant strain was maintained in media supplemented with 50 mg/L hygromycin B (Roche, Indianapolis, IN, USA). BCG was used to explore the biological function of the methyltransferase, HsdM.

### 4.2. Generation of the hsdM Knockout Mutant Strain

Mycobacteriophage-based specialized transduction was used to replace *hsdM* as previously described [18,19]. The upstream and downstream sequences were amplified from BCG genomic DNA, using primer pairs hsdM-LL/hsdM-LR and hsdM-RL/hsdM-RR. Table S4 lists the corresponding primers for the ∆*hsdM* mutant strain, and Figure 1A shows their corresponding positions. The resulting cloned upstream and downstream regions of *hdsM* were ligated with plasmid p0004s (Hsu and Jacobs, unpublished data) digested with *Van91*I. The constructed plasmid p0004-*hsdM* was then linearized with *Pac*I and inserted into the *Pac*I-digested phAE159 (Hsu and Jacobs, unpublished data). A MaxPlax packaging extract (Epicentre Biotechnologies, Madison, WI, USA) was used for phage packaging and the resulting shuttle plasmids were transformed and amplified into *E. coli* HB101 cells. The selected plasmids were electroporated into *M. smegmatis* mc^2^155 for phage propagation. Transduction into individual BCG cells was performed and *hsdM* was replaced with hygromycin-coding gene (*hyg*) (Figure 1), and correct transformants were characterized by PCR using the primer pair, hsdM-InL/hsdM-InR (Table S4). The corresponding complementary strain was constructed as described previously. Briefly, the full-length sequence of *hsdM* was amplified from BCG genomic DNA using primer pair 361-hsdM-F/361-hsdM-R (Table S4), and the PCR product was cloned into the integrating vector, pMV361 [37,38]. The constructed plasmid was then electroporated into the knockout strain, ∆*hsdM*, yielding pMV361-*hsdM/*Δ*hsdM*.

### 4.3. Antibiotic Susceptibility Testing

Growth rates of the *hsdM* mutant strain ∆*hsdM* and its parent strain were compared in 7H9 medium by monitoring the OD_600_ at different time points. Early phase cultures (OD_600_~0.1) were treated with drugs at the indicated concentrations. The OD_600_ values and the number of CFUs were measured at the indicated point times. Experiments were performed in triplicate.

Isoniazid (INH), rifampicin (RFP), streptomycin (STR), ethambutol (EMB), ciprofloxacin (CIP) and ofloxacin (OFX) were obtained from Sigma-Aldrich (Saint Louis, MO, USA). Susceptibility of the mycobacterial strains to these antibiotic drugs was determined on microplates. A modified microplate Alamar Blue assay was performed to examine the mycobacterial susceptibility as previously described [39]. Briefly, approximately 10^5^ cells/well were incubated for 7 days with different drug concentrations at 37°C. The indicator, 0.02% resazurin, was then added to individual samples, and color changes (from blue to pink) were recorded after 48 h. Blue indicated no growth; pink indicated growth. The MIC was defined as the lowest antibiotic drug concentration that prevented the color change from blue to pink. A difference of 4-fold or more indicated a significant difference in the antibiotic susceptibility of bacterial strains.

### 4.4. SMRT Sequencing and Bioinformatics Analysis

Genomic DNA was extracted from *M. bovis* BCG strains using a previously described method. Briefly, pellets were scraped from plates containing each strain into 4 mL DNA buffer (0.3 M Tris, pH 8.0; 0.1 M NaCl; 6 mM EDTA) and vortexed vigorously with 3 mm glass beads. The supernatant was treated with lysozyme solution, extracted in phenol/chloroform/isoamyl alcohol and precipitated to obtain the genomic DNA. Whole-genome sequencing was used, combining the Illumina HiSeq2000 (Illumina Inc., San Diego, CA, USA) and Pacific Biosciences Sequel II (Pacific Biosciences, Menlo Park, CA, USA) sequencing platforms, and the sequence data from the Illumina platform were used to proofread the PacBio assembly sequence. An Illumina paired-end sequencing library was prepared using TruSeq DNA sample prep kits (Illumina Inc.) as per the manufacturer’s instructions. A 20-kb SMRT bell library was prepared from sheared genomic DNA using a 20 kb template library preparation workflow. SMRT sequencing was conducted on a PacBio Sequel II sequencing platform.

The Hierarchical Genome Assembly Process (HGAP.4) algorithm in the SMRT Link (version 9.0.0) was used to assemble the genome. Errors in assembly of the raw sequence reads were corrected with the Quiver algorithm included in the SMRT software package [40]. Standard settings (QV > 30) in the “Base Modification Analysis” protocol included in SMRT Link, version 9.0.0, were used to detect base modifications and sequence motifs. The genomic sequence was uploaded into Rapid Annotation using Subsystem Technology for genome annotation. Functions of the predicted protein-coding genes were then annotated via comparisons with the NCBI-NR and COG databases. Functional gene categories were obtained by searching the Mycobrowser website (https://mycobrowser.epfl.ch/genes, accessed on 12 August 2021).

### 4.5. Hypoxia Survival Experiments

BCG strains were cultured in 7H9 media to OD_600_~0.5. Cultures were inoculated at 1 × 10^6^ CFU/mL in anaerobic bottles and tightly sealed. The headspace ratio of the samples was 0.5, as defined by the latent Wayne model [30], and methylene blue (1.5 mg/L) was used as an indicator of reduced oxygen tension. All samples were prepared in triplicate. The cultures were collected when the indicator turned from blue to colorless. Because the OD_600_ reached 0.2 when the indicator became colorless, we used samples grown aerobically to an OD_600_ of 0.2 as controls.

### 4.6. RNA Isolation and Quantitative Real-Time PCR

Mycobacterial cells were collected by centrifugation at 12,000× *g*, and bacterial pellets were resuspended in TRIzol reagent (Invitrogen, Carlsbad, CA, USA). RNA was then purified following the manufacturer’s instructions. cDNA was synthesized using 5× All-In One RT MasterMix (ABM, Richmond, BC, Canada). The EvaGreen 2× qPCR Master Mix (ABM) was used for quantitative real-time PCR in a Bio-Rad CFX Connect Real-Time System. The amplification conditions were set as follows: initial denaturation at 95 °C for 3 min; then, 40 cycles of 95 °C for 20 s, 60 °C for 20 s, 72 °C for 20 s, ending with a melting curve step of 65 °C to 95 °C. The BCG RNA polymerase sigma factor, *sigA*, was used as a control to normalize gene expression. The 2^–∆∆CT^ method [41] was used to calculate the relative mycobacterial gene expression. Table S4 lists the qRT-PCR primers.

### 4.7. Statistical Analysis

The results were those of three biological replicates. Statistical analyses were performed using unpaired two-tailed *t*-tests in GraphPad Prism 6 or Microsoft Excel. ** *p* < 0.01 and * *p* < 0.05.

## 5. Conclusions

In summary, we explored the biological functions of the MTase, HsdM, in BCG and showed that deleting *hsdM* decreased mycobacterial susceptibility to INH and increased mycobacterial survival under hypoxia in BCG. Using SMRT sequencing, we identified 219 HsdM-methylated genes, including *trcR*, a regulator in mycobacterial latency and reactivation. Our study indicated that HsdM methylase genes alter the corresponding gene transcription and, thereby, alter the cellular redox status, resulting in mycobacterial susceptibility to INH. Our work provides insights into the relationship between DNA methylation and antibiotic susceptibility. Further studies are necessary to confirm whether the observed phenotypes related to HsdM are not just restricted to the used BCG model organism, but also to other *M. bovis* BCG and to virulent *M. tuberculosis* strains.

## Figures and Tables

**Table 1:** N6-methyl-adenine base modifications in sequenced BCG strains.

Strain	Methylated Motif					
	CTCCAG		CACGCAG		GATN_4_RTAC	
	No. of Motifs in Genome	% Motifs Detected	No. of Motifs in Genome	% Motifs Detected	No. of Motifs in Genome	% Motifs Detected
BCG	3834	99.3	806	99.8	674	72.5
Δ*hsdM*	3832	99.1	805	100	676	/
pMV361-*hsdM/*∆*hsdM*	3840	99.3	805	100	676	58.7

Note: “_” represented the methylated sites.

## Data Availability

The data presented in this study are openly available in China National Microbiology Data Center (NMDC) with accession numbers NMDC40013959 (https://nmdc.cn/resource/genomics/sra/detail/NMDC40013959, accessed on 12 August 2021) and NMDC40013960 (https://nmdc.cn/resource/genomics/sra/detail/NMDC40013960, accessed on 12 August 2021).

## Supplementary Materials

The following are available online at https://www.mdpi.com/article/10.3390/antibiotics10111323/s1, Figure S1: The MIC of INH on microplates in BCG, Δ*hsdM* and pMV361-*hsdM/*Δ*hsdM*, Table S1: Methylated HsdM substrates: modification sites located in the gene, Table S2: Methylated HsdM substrates: modification sites located upstream of the gene, Table S3: HsdM-methylated substrates involved in intermediary metabolism and the respiration pathway, Table S4: Primers used in this study.
