# Supplementary material for: The Mycobacterial DNA Methyltransferase HsdM Decreases Intrinsic Isoniazid Susceptibility
Source: Antibiotics (Basel). 2021 Oct 29;10(11):1323. doi: 10.3390/antibiotics10111323 (PMC8614780; doi:10.3390/antibiotics10111323)
Supplement: Supplementary file 1 [file antibiotics-10-01323-s001.zip › supplementary files/Supplementary File-1391552.pdf]

Supplementary Figure

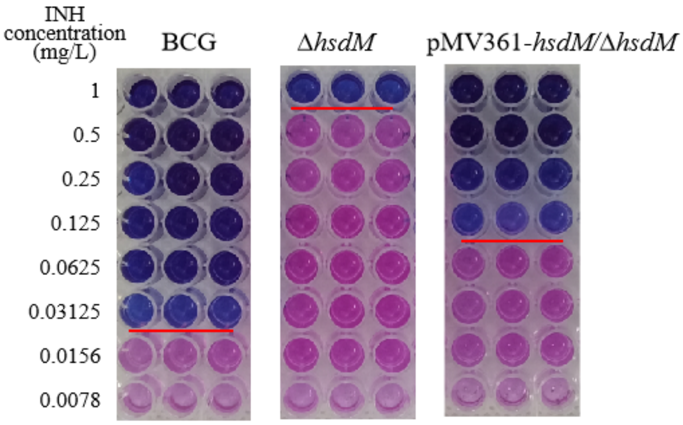

Figure S1. The MIC of INH on microplates in BCG, Δ*hsdM* and pMV361-*hsdM*/Δ*hsdM*. Blue wells indicate no growth owing to drug inhibition and pink wells indicate growth.

Supplementary Table

Table S1. Methylated HsdM substrates: modification sites located in the gene

In a Separated Excel File.

Table S2. Methylated HsdM substrates: modification sites located upstream of the gene

In a Separated Excel File.

Table S3. HsdM-methylated substrates involved in intermediary metabolism and the respiration pathway

In a Separated Excel File.

Table S4. Primers used in this study

In a Separated Excel File.
